# Atherosclerosis: The Culprit and Co-victim of Vascular Dementia

**DOI:** 10.3389/fnins.2021.673440

**Published:** 2021-08-06

**Authors:** Ya-Ting Huang, Fen-Fang Hong, Shu-Long Yang

**Affiliations:** ^1^Department of Physiology, College of Medicine, Nanchang University, Nanchang, China; ^2^Queen Marry College, School of Medicine, Nanchang University, Nanchang, China; ^3^Experimental Center of Pathogen Biology, Nanchang University, Nanchang, China; ^4^Department of Physiology, Fuzhou Medical College, Fuzhou, China

**Keywords:** atherosclerosis, vascular disease, atherosclerosis biomarkers, hypoperfusion, common risk factors

## Abstract

Vascular dementia (VD), a cerebrovascular disease which causes cognitive impairment, is one of the significant factors that affects the quality of senectitude. Atherosclerosis (AS) is a chronic inflammatory syndrome and closely associated with VD. Analyzing the role of AS in VD contribute greatly to its early detection and prevention, but their relationship has not been integrated into a complete network. This review summarizes AS biomarkers as VD predictors for the first time and describes the direct mechanisms of AS causing VD from five aspects: vascular morphogenesis, hemodynamic change, neurovascular unit damage (NVU), oxidative stress, and microRNA (miRNA). Finally, it discriminates the relationship between AS and VD in common risk factors which can be disease or some molecules. In particular, these data imply that the role of AS in VD is not only a pathogenic factor but also a comorbidity in VD. This review aims to bring new ideas for the prediction and treatment of VD.

## Introduction

Atherosclerosis (AS) is a chronic inflammation, and its lesions are often located in large or medium-sized arteries. It is characterized by the accumulation of low-density lipoproteins (LDLs), together with oxidized lipoproteins and cholesterols. The LDLs are engulfed by phagocytes to form foam cells. Subsequent recruited inflammatory cells exacerbate the AS progress (Bayatmakoo et al., 2017). In the end, the continuous narrowing of the lumen caused by gradually expanding atheromatous plaque restricts blood flow.

Vascular dementia (VD) is a type of cognitive impairment contributed by cerebrovascular disease, whose clinical manifestations are memory decline, early gait disorder, incontinence with mood (e.g., depression and apathy), and personality changes (Habeych and Castilla-Puentes, 2015). The WHO estimates there will be about 82 million people with dementia in 2050, twice as many as now (Javanshiri et al., 2018). VD is a fatal subtype of dementia. Due to the incurable nature of the condition, patients have many complications, high frequency of readmission, and poor self-care ability. They almost completely rely on family guardianship and medical service, which takes up extensive social resources, and suffer from heavy psychological and physical burden. As a result, the prevention of VD is a priority. To actively find out the role of AS in VD is conducive to timely prediction and early intervention of VD patients. It also provides ideas and therapeutic targets for the treatment of VD.

According to an experiment by Chen et al., vegetarians with a lower risk of AS also had a lower incidence of cognitive impairment (Chen et al., 2018), which reflects the relationship between blood vessels and cognition, allowing further hypothesis about AS playing a role in VD. Prior trials have shown that AS has a significant influence on VD (Chen et al., 2018; Javanshiri et al., 2018). There are currently two theories about their relationship. One is that AS and VD share common risk factors, which occur at the same time. They are complications of each other and affected by the same molecular mechanism. On account of the earlier onset of AS, some studies set the second standpoint - AS plays an important role in the development of VD, including a direct role and an indirect role in combination with other diseases.

In this review, we address the predictive role of AS in VD, followed by summarizing two existing hypotheses about the role of AS in VD, before ending with perspectives for future research.

## AS Biomarkers

Midlife vascular disease plays a crucial part in the subsequent development of dementia (Gottesman et al., 2017). Hence, studies emphasizing the relationship between AS biomarkers and VD are in great demand. If monitoring AS can indicate VD, it will give a new perspective to VD predictions. Table 1 summarizes nine AS biomarkers, which are classified into five types, namely vascular function, thrombosis, metabolism, lipid, and inflammation, to verify the feasibility of the expectation. Since mild cognitive impairment marks the transition from normal cognition to dementia, this paper incorporates it into the research scope (Camarda et al., 2018).

**TABLE 1:** Summary of the AS biomarkers in VD.

**Classification**	**AS biomarker**	**Reported correlation with VD**	**References**
Vascular function	IMT	Higher midlife IMT has significant association with VD.	Gustavsson et al., 2020
		IMT is associated with cognitive decline and dementia.	Harlé and Plichart, 2015
		Severe IMT has a strong relationship with increased risk of dementia.	Qiu and Fratiglioni, 2015
	FDM	FDM may be the most sensitive factor in predicting cognitive decline.	Tachibana et al., 2016
	ABI	Low ABI is associated with cognition in the presence of neurodegeneration.	Shaik et al., 2017
		Low ABI may manifest susceptibility of cognitive impairment and will increase during senescence.	Qiu and Fratiglioni, 2015
	CAC	High CAC is markedly associated with increased risk of dementia.	Fujiyoshi et al., 2017
		Higher CAC was faintly correlated with inferior executive function.	Suemoto et al., 2017
		Among white women, low CAC score indicates a significantly reduced risk of dementia.	Kuller et al., 2016
		There was no significant correlation between CAC score and WMH, but CAC and WMH co-influenced cortical thinning.	Lee et al., 2016
Thrombosis	Atherosclerotic plaque	The presence of CP is not related to VD, and only high plaque scores are significantly correlated with VD.	Gustavsson et al., 2020
		Flimsy CP increases the risk of cortical CMIs which play an important role in VD.	Takasugi et al., 2019
		Descending aorta plaque is a risk marker for brain injury and aging.	Aparicio et al., 2017
		The strain instability of atherosclerotic plaque predicted vascular cognitive decline in asymptomatic patients directly.	Dempsey et al., 2018
		CP helps to assess the individual risk of dementia and the risk of mild cognitive impairment progression.	Harlé and Plichart, 2015
		The presence of increasing CP is associated with an increased risk of dementia	Qiu and Fratiglioni, 2015
		In the elderly, CP is a VD independent predictor.	Carcaillon et al., 2015
Metabolism	Serum cystatin C and microalbuminuria	Among the middle-aged, advanced cognitive function was notably and inversely correlated with Serum cystatin C and microalbuminuria.	Kono et al., 2017
	Cholesterol	Midlife cholesterol can forecast dementia which has vascular features.	Rantanen et al., 2017
Inflammation	MIC-1/GDF15	MIC-1/GDF15 is inversely proportional to cognitive function and can be a marker of early WM damage.	Jiang et al., 2015
	IL-6	IL-6 had a significant effect on dementia in patients with vascular risk factors.	Miwa et al., 2016
		Il-6 plasma levels were negatively correlated with VD.	Bǎdescu et al., 2016

*VD, Vascular dementia; AS, Atherosclerosis; IMT, intima media thickness; FDM, Flow-mediated dilation; ABI, Ankle-brachial index; CAC, Coronary artery calcium; CP, carotid plaque; CMIs, cortical cerebral microinfarcts; WMH, white matter hyperintensities; MIC-1/GDF15, Macrophage inhibitory cytokine-1; IL-6, Interleukin-6.*

Intima media thickness (IMT) is a pivotal AS biomarker which is measured by ultrasonography. Higher midlife IMT has prominent relevance to VD and a strong relationship with increased risk of dementia (Harlé and Plichart, 2015; Qiu and Fratiglioni, 2015; Gustavsson et al., 2020). So IMT is a relatively reliable marker. Flow-mediated dilation (FMD) generally represents the human endothelial vasodilating function. It may be the most sensitive predictor of vascular function in cognitive decline and the early stage of AS. Low FMD is correlated with VD. Patients with VD exhibit a certain amount of endothelial dysfunction which indicates the initiation of AS (Tachibana et al., 2016; Karlsson et al., 2017). Ankle-brachial index (ABI) is a quick, simple, and inexpensive measurement considered as a biomarker of AS; low ABI is associated with cognition in the presence of neurodegeneration and may manifest higher susceptibility of cognitive impairment during senescence (Kono et al., 2017; Shaik et al., 2017). Coronary artery calcium (CAC) is a powerful quantitative biomarker of AS. However, the association between CAC and dementia is controversial, some researchers support the idea that the risk of dementia is associated with the CAC score (Kuller et al., 2016; Fujiyoshi et al., 2017), while some are negative (Lee et al., 2016). A cross-section study conducted by Suemoto et al. suggested that higher CAC is faintly correlated with inferior executive function (Suemoto et al., 2017). The differences among the various experimental results should be noted. More research is needed to determine whether CAC is associated with VD or not. Likewise, atherosclerotic plaque is a biomarker of AS, but its predictive role in VD depends on plaque score, such as the stability, numbers, and location of the plaque. Only high plaque score is significantly associated with VD (Qiu and Fratiglioni, 2015; Aparicio et al., 2017; Dempsey et al., 2018; Takasugi et al., 2019; Gustavsson et al., 2020). It is also proven to be one of VD independent predictors in the elderly (Carcaillon et al., 2015; Dempsey et al., 2018). However, serum cystatin C and microalbuminuria can be applied at an earlier age. Advanced cognitive function is notably and negatively correlated with the two biomarkers among the middle-aged (Kono et al., 2017). Cholesterol abnormity is an important feature and one of the pathogenic mechanisms of AS. Some scholars have conducted studies on the relationship between midlife abnormal cholesterol and VD. Finally, they concluded that patients with VD in old age have higher midlife cholesterol (Rantanen et al., 2017). Macrophage inhibitory cytokine-1 (MIC-1/GDF15) and interleukin-6 (IL-6) are both inflammatory markers linked with AS, which are inversely proportional to VD when used as biomarkers for VD (Jiang et al., 2015; Bǎdescu et al., 2016; Miwa et al., 2016).

When using AS biomarkers for prediction, clinicians should consider multiple parameters, and combine more than one biomarker to improve the accuracy of VD prediction. According to AS progress, different AS biomarkers can be selected for detection. For example, FDM can be used to predict in the early stage of AS, while microalbuminuria can be used in the later stage (Tachibana et al., 2016; Kono et al., 2017). However, it should be noted that the significant correlation between the AS biomarkers and VD does not equal to a causality relationship between them.

## The Mechanism Underlies AS Causing VD

**FIGURE 1:**
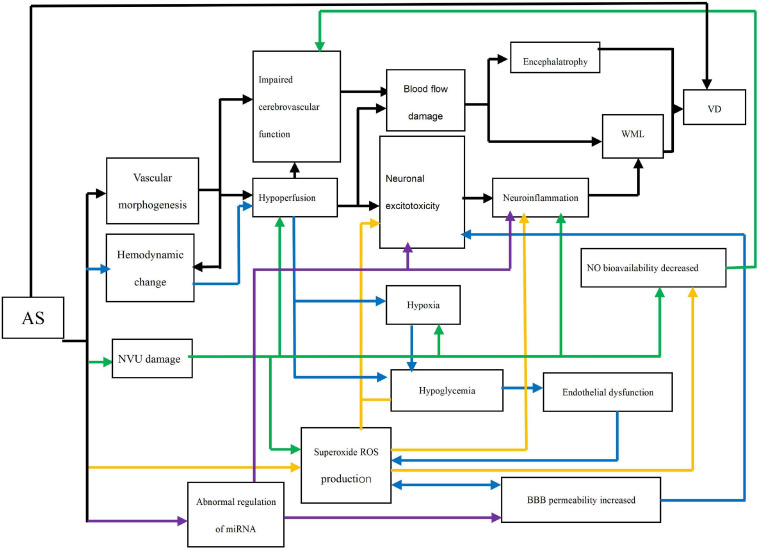
Direct networks of AS and VD. AS: Atherosclerosis; Impaired cerebrovascular function: Endothelial relaxation and cerebrovascular automatic regulation; Neuronal excitotoxicity: Oligodendrocyte death and activation of microglia and astrocytes; WML: White matter lesion (e.g., demyelination); VD: vascular dementia; NO: Nitric oxide; ROS: Reactive oxygen species; NVU: neurovascular unit; BBB: Brain-blood barrier. Black line: The path of vascular morphogenesis; Blue line: The path of hemodynamic change; Green line: The path of NVU damage; Yellow line: The path of oxidative stress; Purple line: The path of abnormal regulation of miRNA.

Middle-aged vascular risk factors affect cognitive function and dementia in later life through different mechanisms. Ample evidence suggests that AS may play a direct role in the progress of VD through a complex network (Figure 1). AS can lead to VD through five pathways, which are vascular morphogenesis, hemodynamic change, neurovascular unit (NVU) damage, Oxidative stress, and microRNAs (miRNAs). These pathways are interconnected and interact together to cause VD.

### Vascular Morphogenesis

The changes in vascular morphology included stenosis, occlusion, and intima-media thickening, which are all characteristics of AS (Alhusaini et al., 2018). Occlusion of blood vessels can chronically disrupt central hemodynamics, cerebral perfusion, and cerebrovascular function. The cerebrovascular functions’ injury includes endothelium-dependent diastolic function and cerebrovascular auto-regulation function injury, namely vascular system damage (Aparicio et al., 2017; Ogoh, 2017). The most direct result of impaired cerebrovascular function is cerebral blood flow (CBF) change. The association between carotid AS and encephalatrophy provides evidence that decreased CBF may lead to encephalatrophy (Moroni et al., 2016). Brain atrophy is a precursor to VD (Tariq and Barber, 2018). Likewise, chronic hypoperfusion causes blood flow damage and neuroinflammation by activating microglia cells, eventually resulting in white matter lesion (WML) and neurodegeneration. WML leads to cognitive impairment and ultimately to VD (Bǎdescu et al., 2016; Lim et al., 2019).

### Hemodynamic Change

AS alters stable blood flow pattern, which triggers chronic hypoperfusion (Nie et al., 2018). When the brain is under chronic ischemia, the small cerebral arteries expand to the maximum extent due to the compensatory mechanism, resulting in impaired cerebral vascular function (Nagata et al., 2016). Even transient ischemia will lead to hypoxia and hypoglycemia, stimulating neuronal excitotoxicity. The core of this toxicity is astrocyte activation. Intracellular mitochondrial autophagy increases intracellular calcium ion and activates glutamate activation accompanied by ATP release. In severe cases, neurons die (Lénárt et al., 2016; Shabir et al., 2018). In the experiment by Nie et al., the loss of neurons in the hippocampus of bilateral implantation of the shear stress modifier mice were observed with a decrease in glucose intake. The evidence demonstrates that a hypoxic environment also causes low sugar level (Nie et al., 2018). Low glucose level rapidly leads to endothelial dysfunction or induces endothelial cell mitochondrial autophagy, which results in nitric oxide (NO) bioavailability decrease, mitochondrial reactive oxygen species (ROS) production increase, and vascular function damage (Tanner et al., 2017). Of important note is that only elastic arterial lesions are associated with VD. VD patients have some degree of endothelial dysfunction, which may be explained by the arterial pulse system (Geijselaers et al., 2016). The increase of mitochondrial ROS and brain–blood barrier (BBB) permeability form a vicious cycle. They jointly cause the death of oligodendrocytes and the activation of microglia and astrocytes (Lim et al., 2019). Following the trajectory mentioned above, the series of processes induce WML and eventually form VD.

### NVU Damage

The NVU links the nervous and circulatory systems both functionally and structurally in the brain in response to the isolation of BBB. NVU damage leads to chronic hypoperfusion, hypoxia, excessive proinflammatory factor production, ROS production, and reduced NO utilization (Ramalho et al., 2018; Shabir et al., 2018). NO is a substance that stimulates vasodilation. The decrease in NO utilization indicates that vascular function is impaired. Importantly, CBF regulation in the aged more depends on this pathway (Karlsson et al., 2017). This may be the reason why they are more susceptible to VD. Meanwhile, the response of cerebral circulation to carbon dioxide (CO_2_) is also damaged. CO_2_ controls the increase and decrease of CBF by controlling the contraction and relaxation of cerebral blood vessels under normal conditions (Nagata et al., 2016). These damages further disrupt blood flow. The subsequent development of impaired blood flow is as previously described.

### Oxidative Stress

AS can directly trigger ROS production through the foam cells in atherosclerotic plaques, and then impair the cerebral vascular function through low bioavailability of NO or lead to WML by triggering neuroinflammation (Karlsson et al., 2017). It means that ROS can be secreted not only by other pathways but also directly by AS, which greatly increases the perniciousness of ROS. Paraoxonase-1(Pon-1) is an enzyme that exists in high density lipoprotein (HDL). When the activity of arylesterase (ARE), which catalyzes the hydrolysis of non-phosphorous ester in the Pon-1 mechanism, is reduced, the enzyme’s anti-atherosclerosis effect is impaired through antioxidant damage, thus promoting the AS process. The serum ARE activity in VD was also significantly decreased (Castellazzi et al., 2016). This provides theoretical support for AS directly causing VD through oxidative stress.

### Abnormal Regulation of miRNA

miRNAs are non-coding RNAs and play an important role in the progression of AS (Feinberg and Moore, 2016). Furthermore, miRNAs are involved in the expression of genes related to neuronal excitotoxicity, neuroinflammation, and BBB permeability increase (Tiedt and Dichgans, 2018). In AS, abnormal regulation of miRNAs contributes to the development of VD like the butterfly effect. miRNA-124 is a representative example. During the process of AS, miRNA-124 is down-regulated (Zhang et al., 2017). However, due to the variation, the gene regulatory procedures involved in it will develop toward a harmful direction such as excitatory toxicity, inflammation, and BBB destruction, eventually following the respective pathway to VD (Tiedt and Dichgans, 2018).

### The Effect of Lesion Location

Different anatomical lesion locations of AS influence the mechanisms of AS causing VD. Here, we take the example of intracranial atherosclerosis (ICAS) and extracranial atherosclerosis (ECAS). The lesions of ICAS occur in the arteries surrounded by the skull and dura mater or located in the subarachnoid space (Qureshi and Caplan, 2014). This means that ICAS can shorten the above five pathways by taking advantage of its location and causes blood flow damage faster than ECAS. Interestingly, ICAS can lead to VD without other mechanisms (Simonetto et al., 2019). This is supported by a higher load of WMH surrounded by vulnerable atherosclerotic plaques in the cerebral hemisphere (Altamura et al., 2016). However, ECAS tends to cause VD through vascular morphogenesis and hemodynamic changes (Kim and Caplan, 2016). Since the descending aorta blood flow is separated from the cerebral circulation, we chose representative descending aorta AS to illustrate the point. In the study by Aparicio et al., descending aortic AS can generate significant encephalatrophy through vascular stenosis and vascular pulsating pressure increase (Aparicio et al., 2017). This is consistent with the vascular morphogenesis and hemodynamic change pathways mentioned above. It demonstrates that although ICAS and ECAS share five common pathogenetic mechanisms, they still have differences.

### Specific Direct Mechanism: Mediated by Stroke

In addition to the fact that AS is directly associated with VD through its mechanisms, the two can be linked by stroke as a mediator. Because many reports only investigate the relationship between AS and stroke, and stroke can directly lead to VD, this paper describes this situation separately (Shabir et al., 2018). Stroke is divided into ischemic stroke and hemorrhagic stroke. AS plays a pathogenic role in stroke, and carotid AS leads to ischemic stroke through the athero-embolic mechanism. The incidence about the recurrence rate of stroke is gradually increasing associated with the increase of AS risk factors and the number of affected vessels (Heldner et al., 2018). VD may be caused by one or more stroke risk factors (Tariq and Barber, 2018). This provides theoretical support for VD mediated by stroke from AS. Herein, the relationship between AS and stroke will be analyzed from the perspectives of genes and molecules.

AS and stroke have a genetic correlation (Franceschini et al., 2018). For example, SH2B3 gene mutation leads to the deficiency of the LNK protein translated by it, which will aggrandize platelet production and activity, thereby accelerating AS. Furthermore, the gene is a risk site for stroke in GWAS (Dichgans et al., 2019). While the causal connection between the gene variations and the development of the two diseases is not elaborate, inflammatory cytokines released by AS can alter gene expression in peripheral blood cells, which suggests that AS may cause the gene mutations and increase the risk of stroke (Ferronato et al., 2019). The hope is that more research will focus on the sequence of genetic mutations and the two diseases in the future.

MCP-1 is a pro-inflammatory factor that plays a significant role in AS and stroke. Its obligation in AS is to recruit monocytes into the nidus. Therefore, the increased level of this factor will aggravate the condition of AS. Research shows MCP-1 level increase for genetic factors compounds the risk of stroke. It should be emphasized that MCP-1 levels are associated with the risk of ischemic stroke, not hemorrhagic stroke. This is consistent with the atherogenic characteristics of stroke (Georgakis et al., 2019). This means there is a close affinity between AS and stroke, people with a genetic variant that causes over normal MCP-1 level in AS have higher stroke risk.

## Common Risk Factors of AS and VD

The occurrence of disease is not caused by a single factor, but by the combination of multiple factors in most cases, which is determined by the diversity of pathogenesis. Therefore, as the common risk factors of AS and VD, it may play a predominant or auxiliary role in the relationship between them. In parallel, the role of AS in VD is double status, which means it can promote VD, directly or indirectly, and can be a co-victim of VD. The common risk factors can be a disease or hereditary factor. Since the pathogenesis of AS and VD overlaps to a certain extent, it is undeniable that among these common risk factors, AS and VD may have a potential causality correlation. In the future, more studies are needed to prove whether these common factors independently lead to AS and VD. By summarizing common risk factors, more associations between AS and VD can be found, which can provide more evidence for clinicians’ diagnosis.

### Chronic Kidney Disease

There is vascular comparability between kidney and brain, so the pathogenesis of kidney disease and VD is similar (Saji et al., 2016). VD is the main cause of cognitive impairment in patients with chronic kidney disease (CKD) (Pépin and Villain, 2020). CKD produces uremic toxins, leads to BBB destruction, and causes neuron damage when the toxin enters the brain. While AS also increases the permeability of BBB, accelerating the ingress of uremic toxins and other hazardous substances into the brain and engendering brain damage (Chelluboina and Vemuganti, 2019). This means AS can not only play a dominant role in the pathogenesis of VD, but also act as an auxiliary factor in the development of VD caused by CKD. In this instance, AS is not a necessary condition to cause disease, but accelerates disease progression.

### Gut Microbiota

The gut microbiota (GM) communicates with the brain via hormones, nerves, and the immune system. It is an important component of the gut-brain axis, and studies have shown that GM can ameliorate the disease development of VD, which provides theoretical foundations for the correlation between GM and VD (Liu et al., 2015). There is GM DNA in Atherosclerotic plaques, suggesting that GM may place a premium on AS and/or participate in the progression of AS through inflammatory responses, lipids, and metabolites. AS and VD can be severally induced by GM. In addition, Helicobacter pylori (Hp) positive patients in VD have graver AS, which supports GM, AS, and VD to form a triangle. Furthermore, the GM expresses more opportunistic bacteria in stroke patients (Xu et al., 2016; Li et al., 2018). Consequently, it is inconclusive whether the severity of AS affects the susceptibility of GM or GM enhances the aggressiveness of AS. Further prospective research is needed to better ascertain the role of GM amongst AS in VD.

### Hereditary Factor

Apolipoprotein E (ApoE) ɜ4 is an allele and produces ApoE. It is a plasma protein involved in lipid metabolism and cholesterol transport (Yin et al., 2012). Carriers of ApoE ɜ4 are strongly associated with VD, and can increase the risk of VD. However, the data on the relationship between ApoE ɜ4 and VD should be used circumspectly for some data suggesting that ApoE ɜ4 and VD are irrelevant (Davidson et al., 2006). The allele is also correlated with higher serum cholesterol levels and the severity of AS in the early stages of youth (Hixson, 1991). β- and γ-secretases lyse the amyloid precursor protein and generate Amyloid beta 1-40 (Abeta40) peptides. The staple amyloid protein in human AS is Abeta40 (Stamatelopoulos et al., 2015). When Abeta40 is further cleaved, the products released are connected with the dominating pathological mechanism of VD (Chen et al., 2019). This may be the progression of ApoE ɜ4 participating in AS and VD. In this case, since AS and VD are both victims, the difference in the onset time of these two diseases may be due to the different course of disease.

## Discussion

This review attempts to identify the role of AS in VD, confirms its irreplaceable role in VD, and provides theoretical support for the prediction of VD in the future. The biomarkers of AS were summarized and discussed for the first time, which demonstrates that it makes a significant contribution to the prediction of VD and provides a reference for clinicians to select the best VD predictor at various stages of AS development. Then, according to the direct-action mechanisms of AS on VD, we analyze them from five pathways: vascular morphogenesis, hemodynamic changes, NVU damage, oxidative stress, and miRNA. These deleterious mechanisms synergistically cause the onset of VD pathogenesis. The last topic, which explores the common risk factors of AS and VD, is intricate and attempts to find out other roles of AS in VD. It is concluded that, in addition to being a pathogenic factor, AS can be a comorbidity of VD. To determine the causality between AS and VD, large-scale, fully equipped clinical trials and repeated measurements over longer follow-up periods will be necessary in the future.

Although momentous progress has been made in the relationship between AS and VD so far, there are many deficiencies. For example, the starting point about chronic diseases is still misty, with the coincidence degree of pathogenic mechanism too high between AS and other diseases among the common risk factors, which increases difficulties in the research of the relationship, and the fuzziness of causal relationship may cause biased results. Research about genes may be the emphasis in the future owing to the development of precision medicine and the enhancement of personalization.

In conclusion, identifying the role of AS in VD deeply may not only reveal new therapeutic targets but also improve risk stratification in VD prevention. The accurate prediction of high-risk groups can also greatly save social resources and reduce panic among the population.

## Author Contributions

Y-TH designed and conceptualized the study and drafted the manuscript for intellectual content. S-LY played a major role in the acquisition of date, provided funds, and revised the manuscript for intellectual content. F-FH revised the manuscript for intellectual content and provided funds.

## Conflict of Interest

The authors declare that the research was conducted in the absence of any commercial or financial relationships that could be construed as a potential conflict of interest.

## Publisher’s Note

All claims expressed in this article are solely those of the authors and do not necessarily represent those of their affiliated organizations, or those of the publisher, the editors and the reviewers. Any product that may be evaluated in this article, or claim that may be made by its manufacturer, is not guaranteed or endorsed by the publisher.
